# Study of the Effects of Deuterium-Depleted Water on the Expression of GLUT4 and Insulin Resistance in the Muscle Cell Line C2C12

**DOI:** 10.3390/biomedicines12081771

**Published:** 2024-08-06

**Authors:** Masumi Kondo, Kaichiro Sawada, Yosuke Matsuda, Makiko Abe, Noriyuki Sanechika, Yumi Takanashi, Yoshitaka Mori, Moritsugu Kimura, Masao Toyoda

**Affiliations:** 1 Division of Nephrology, Endocrinology and Metabolism, Department of Medicine, Tokai University Hachioji Hospital, 1838 Ishikawacho, Hachioji 192-0032, Japan; masumi.k@tokai.ac.jp (M.K.); makiko8031@yahoo.co.jp (M.A.); 2 Division of Nephrology, Endocrinology and Metabolism, Department of Medicine, Tokai University School of Medicine, 143 Shimokasuya, Isehara 259-1193, Japan; sawada.kaichiro.f@tokai.ac.jp (K.S.); yosuke199260@gmail.com (Y.M.); sanechika.noriyuki.c@tokai.ac.jp (N.S.); takanashi.ym@tokai.ac.jp (Y.T.); mori.yoshitaka.k@tokai.ac.jp (Y.M.); km1689@tokai.ac.jp (M.K.)

**Keywords:** deuterium-depleted water, GLUT4, insulin resistance, diabetes

## Abstract

Deuterium-depleted water (DDW) is used in the treatment of many diseases, including cancer and diabetes. To detect the effect of DDW on gene expression and activation of the insulin-responsive transporter GLUT4 as a mechanism for improving the pathology of diabetes, we investigated the GLUT4 expression and glucose uptake at various concentrations of DDW using the myoblast cell line C2C12 differentiated into myotubes. GLUT4 gene expression significantly increased under deuterium depletion, reaching a maximum value at a deuterium concentration of approximately 50 ppm, which was approximately nine times that of natural water with a deuterium concentration of 150 ppm. GLUT4 protein also showed an increase at similar DDW concentrations. The membrane translocation of GLUT4 by insulin stimulation reached a maximum value at a deuterium concentration of approximately 50–75 ppm, which was approximately 2.2 times that in natural water. Accordingly, glucose uptake also increased by up to 2.2 times at a deuterium concentration of approximately 50 ppm. Drug-induced insulin resistance was attenuated, and the glucose uptake was four times higher in the presence of 10 ng/mL TNF-α and three times higher in the presence of 1 μg/mL resistin at a deuterium concentration of approximately 50 ppm relative to natural water. These results suggest that DDW promotes GLUT4 expression and insulin-stimulated activation in muscle cells and reduces insulin resistance, making it an effective treatment for diabetes.

## 1. Introduction

Deuterium (D) is a stable isotope of hydrogen (H) with a mass of 2. Natural water contains approximately 150 ppm HDO, and differences in its chemical and physical properties are known to have various effects on living organisms. Its involvement in cancer has attracted attention since the last century. Specifically, it has become clear that high deuterium concentrations promote the growth of cancer cells, whereas low deuterium concentrations inhibit the growth of cancer cells and cause tumor regression [1,2,3]. As an application for cancer treatment, various animal experiments and clinical studies have reported that drinking deuterium-depleted water (DDW) suppresses the expression of cancer genes and causes tumor regression, indicating the possibility that DDW can be used as a safe anticancer drug [4,5,6,7].

It has been proposed that the mechanism of the anticancer effect of DDW involves the function of Na+/H+ transporters and H+-ATPase, as well as the mitochondrial NADPH synthesis pathway, but much remains unknown [8,9].

Studies on cancer treatment with DDW have revealed that in cancer patients with diabetes, DDW improves the condition of diabetes, as well as the effects of cancer treatment. It has been reported to affect glucose metabolism, and recent studies have shown that it affects the activity of glucose transporters (GLUTs), which control glucose uptake [10,11,12]. GLUT4 is a protein expressed in muscle and fat cells. The uptake of sugar is regulated by insulin. This molecule is directly involved in insulin resistance, which manifests itself as advanced diabetes. In normal cells, GLUT4 translocates to the cell membrane upon insulin stimulation to take up glucose; however, insulin resistance is thought to occur when GLUT4 expression is reduced or membrane translocation is inhibited [13,14,15,16].

Recent studies using animal models of diabetes have reported that drinking DDW increases the effect of insulin administration and promotes the membrane translocation of GULT4, indicating the possibility of using DDW as a treatment for diabetes [11,12].

To clarify the mechanism by which DDW improves the pathology of diabetes, we investigated the expression and activation of GLUT4 by DDW in a muscle cell line. Furthermore, we investigated glucose uptake under DDW and measured the effect of DDW on insulin resistance induced in cultured cells, demonstrating the possibility that DDW could significantly improve diabetes and demonstrating the contribution of in vitro systems toward exploring this mechanism.

## 2. Materials and Methods

Deuterium-depleted water (DDW) was provided by Super Light Water Co., Ltd. (Tokyo, Japan). The deuterium concentration was measured using the δ-D equilibration method with an Isotope Ratio Mass Spectrometer (model DELTA Plus XL, Thermo Fisher Scientific, Waltham, MA, USA) [17]. The deuterium concentration in DDW was 15–20 ppm.

Regarding the cell culture, the mouse myoblast cell line C2C12 was obtained from the JCRB Cell Bank (Osaka, Japan). Cells were cultured in DMEM (high-glucose) (Invitrogen, Carlsbad, CA, USA) supplemented with 10% fetal calf serum, 1× GlutaMAX-1, 50 U/mL penicillin, and 50 mg/mL streptomycin in tissue culture plates in a humidified atmosphere of 95% air/5% CO_2_ at 37 °C. After confluence, the medium was replaced with medium supplemented with 2% horse serum to promote myotube differentiation, and the cells were cultured for 10 days or 2 weeks [18,19,20]. At 48 h before harvest, the medium was replaced with medium adjusted to each deuterium concentration using DDW. To determine the optimal deuterium concentration, media with deuterium concentrations ranging from 25 ppm to 150 ppm were used. Insulin (10 μg/mL) was added 30 min before harvest. Insulin resistance was induced in cells by adding 10 ng/mL tumor necrosis factor-α (TNF-α) or 1 μg/mL resistin in culture medium for 24 h.

For the quantitative real-time polymerase chain reaction (qRT-PCR), complimentary DNAs were prepared from cultured cells using a SuperPrep II Cell Lysis and RT kit for qPCR (Toyobo, Osaka, Japan). The reaction mixture was prepared according to the manufacturer’s instructions (TaqMan Gene Expression Assays; Thermo Fisher Scientific) and contained primers and probes for mouse GLUT4 (Assay ID: Mm00436615_m1) and 18S ribosomal RNA endogenous control (Thermo Fisher Scientific). PCR was performed using an ABI StepOne Plus (Thermo Fisher Scientific). Data were analyzed using the comparative Ct method, and GLUT4 mRNA was expressed relative to that of the endogenous control [21].

For Western blotting, cells were lysed using xTractor buffer (Takara Bio, Kusatsu, Japan) with a ProteoGuard protease inhibitor (Takara Bio) and cryonase nuclease (Takara Bio). Sodium dodecyl sulfate–polyacrylamide gel electrophoresis (SDS-PAGE) was performed with NuPAGE 4–12% Bis-Tris gel (Thermo Fisher Scientific), and proteins were transferred to a PVDF membrane with an iBlot2 dry blotting system (Thermo Fisher Scientific). After blocking with 5% BSA in Tris-buffered saline (TBS), the PVDF membrane was treated with a mouse monoclonal antibody against mouse GLUT4 (ab238661; Abcam, Cambridge, UK), washed with TBS containing 0.05% Tween 20, and treated with horseradish peroxidase-conjugated anti-mouse IgG antibody. After washing, the reaction was visualized using EzWestBlue W solution (ATTO Co., Ltd., Tokyo, Japan) [22]. Image analysis was performed using the ImageJ software (version. 1.54h) [23].

For membrane fraction analysis, membrane fractions were isolated from the cells using a ProteoExtract Transmembrane Protein Extraction Kit (Merck, Darmstadt, Germany) [24]. Membrane proteins from cells cultured in media with various concentrations of DDW were prepared as described in the kit manual, and Western blotting was performed to detect GLUT4, as described above. 

For the glucose uptake assay, the glucose uptake in the cells was detected using the Glucose Uptake Assay Kit—Green (DOJINDO LAB. Kumamoto, Japan), according to the manufacturer’s instructions [25]. Fluorescence-conjugated glucose was photographed with a fluorescence microscope, and the fluorescence intensity was measured with a fluorescence spectrophotometer SpectraMax i3 (Molecular Devices, San Jose, CA, USA).

Re statistics, R (version. 4.3.2) was used for the statistical calculations [26]. The results are presented as the mean ± standard deviation (SD) of at least three samples. Comparisons between two groups were performed using Student’s *t*-test. Statistical significance was set at *p* < 0.05.

## 3. Results

### 3.1. Promoting Effect of DDW on the Expression of GLUT4

**Figure 1:**
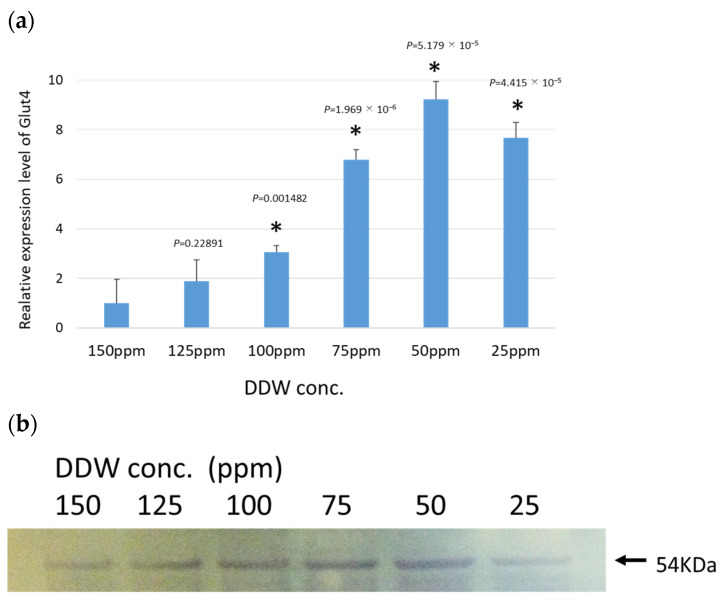
Effect of DDW on the GLUT4 expression in myodifferentiated C2C12 cells. The expression of GLUT4 was examined at the gene and protein levels in the fully differentiated myotubes of C2C12 cells cultured in media adjusted with various concentrations of DDW for 48 h and stimulated with insulin for 30 min before harvest. (**a**) Detection of the expression of GLUT4 by qRT-PCR. The graphs show the relative values compared with those of the natural water medium (150 ppm). Each sample number was ≥3. * Significant difference compared with the natural water medium (150 ppm), *p* < 0.05. (**b**) Diagram of GLUT4 protein expression detected by Western blotting. The arrow indicates the position of the GLUT4 band (54 KDa).

To investigate the effect of DDW on GLUT4 expression, mouse C2C12 cells were differentiated into myotubes, and the effect of DDW on GLUT4 expression was measured. Fully differentiated myotube C2C12 cells were cultured in media adjusted to various deuterium concentrations for 48 h and harvested after stimulation with insulin for 30 min, and the amount of GLUT4 mRNA was compared. A significant increase in the amount of GLUT4 mRNA was observed in the medium with a lower deuterium concentration than in cells cultured in a medium containing natural water (deuterium concentration of 150 ppm) (Figure 1a). This increase was also detected at a deuterium concentration of 125 ppm but was clear at 100 ppm or less, with a peak at 50 ppm. At this time, the expression was approximately nine times that in the natural water medium. When the deuterium concentration was further reduced, the amount of expression decreased but remained higher than that of natural water.

The promoting effect of DDW on the expression of GLUT4 was also confirmed at the protein level (Figure 1b). Western blotting showed that GLUT4 protein, which is difficult to detect in natural water, increased as deuterium concentration decreased, with the maximum amount being at 50–75 ppm deuterium, corresponding to the results at the mRNA level. 

These results demonstrated that DDW promoted the expression of GLUT4 in muscle-differentiated C2C12 cells.

### 3.2. Activation of GLUT4 by DDW

**Figure 2:**
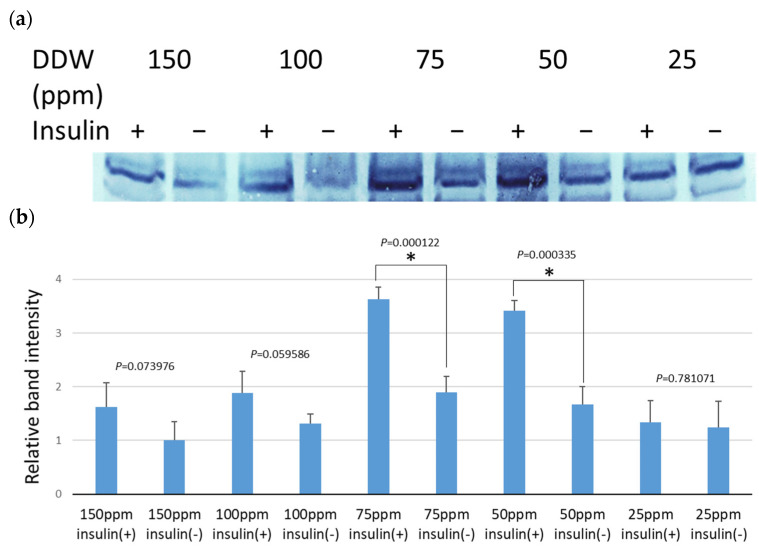
Effect of DDW on the membrane translocation of GLUT4. Fully differentiated C2C12 myotubes were cultured for 48 h in media containing various concentrations of DDW. After insulin stimulation to induce the membrane translocation of GLUT4, the cells were harvested and membrane proteins were extracted. GLUT4 protein was detected and quantified using Western blotting. (**a**) Western blotting of the membrane protein fraction; (**b**) GLUT4 bands were quantified using ImageJ. Values relative to those in cultures without insulin in natural water medium (150 ppm) are shown. The sample size was four. * Significant difference between the presence and absence of insulin in DDW medium at the same concentration (*p* < 0.05).

Before insulin stimulation, intracellular GLUT4 accumulates around the nucleus; however, its function is activated by translocation to the cell membrane upon insulin stimulation. To investigate the effect of DDW on GLUT4 activation, fully myotube-differentiated C2C12 cells were cultured for 48 h in media containing various concentrations of DDW, and the GLUT4 in the cell membrane fraction was detected by Western blotting (Figure 2).

Even without insulin stimulation, a decrease in deuterium concentration promoted the membrane translocation of GLUT4, reaching a maximum of approximately twice that of natural water at 50–75 ppm. Insulin stimulation in natural water medium induced a 1.6-fold increase in GLUT4 translocation to the membrane relative to the non-stimulated group, while DDW medium further increased GLUT4 translocation. The peak occurred at a deuterium concentration of 50–75 ppm, and approximately 3.5-fold more GLUT4 was translocated to the membrane relative to non-insulin-stimulated natural water medium. This amount was approximately 2.2-fold that of non-insulin-stimulated DDW medium at the same concentration. These results demonstrated that DDW promotes GLUT4 activation through membrane translocation in muscle-differentiated C2C12 cells.

### 3.3. Effect of DDW on Glucose Uptake and Insulin Resistance

As shown above, DDW promoted the expression and activation of GLUT4; therefore, we observed its effect on the uptake of fluorescence-conjugated glucose into myotube cells.

**Figure 3:**
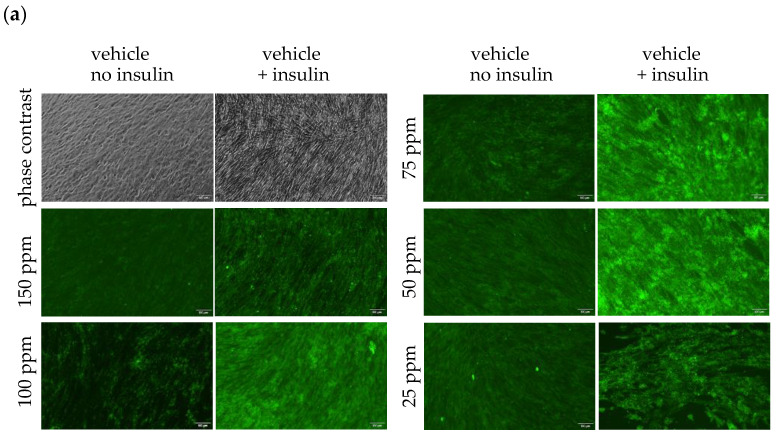

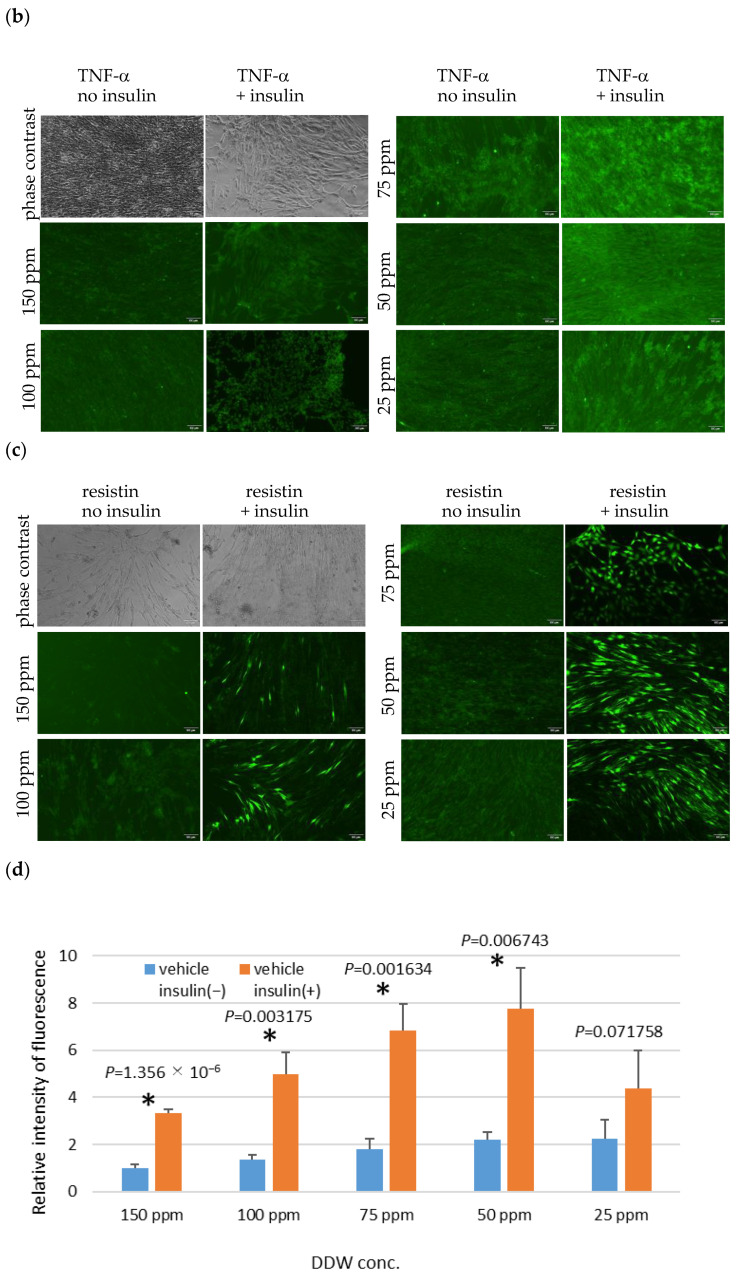

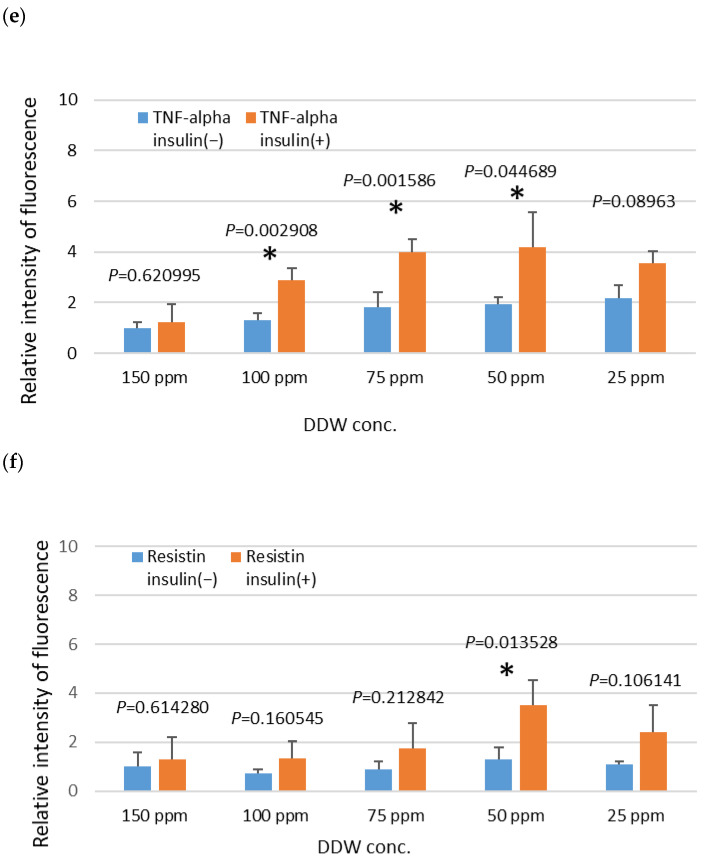
Glucose uptake in myotube-differentiated C2C12 cells. Fully myotube-differentiated C2C12 cells were cultured in a medium containing various concentrations of DDW for 48 h, after which the uptake of fluorescence-conjugated glucose was observed. TNF-α and resistin were added 24 h before harvesting, whereas insulin and fluorescence-conjugated glucose were added 30 min before harvesting. Scale bar: 100 μm. (**a**–**c**) Images of C2C12 glucose uptake. From the left, first and third rows indicate no insulin added; second and fourth rows indicate insulin added. The upper left two images are phase-contrast images of cells cultured in natural water medium (150 ppm) with and without insulin, respectively. The others are fluorescence observations. (**a**) Cells without induction of insulin resistance, (**b**) cells treated with 10 ng/mL TNF-α, and (**c**) cells treated with 10 μg/mL resistin. (**d**–**f**) Fluorescence intensity was measured, and the relative values to culture in natural water medium (150 ppm) without insulin were graphed. The sample size was four. * Significant difference (*p* < 0.05) between the presence (orange bar) and absence (blue bar) of insulin added to DDW medium at each concentration. (**d**) Cells without the induction of insulin resistance, (**e**) cells supplemented with 10 ng/mL TNF-α, and (**f**) cells supplemented with 10 μg/mL resistin.

As a result, in a natural water medium, glucose uptake increased by insulin stimulation by approximately 3.3 times compared with that without insulin stimulation. In DDW medium, even without insulin stimulation, glucose uptake increased with decreasing deuterium concentration, increasing by approximately 2 times at 25–75 ppm. Glucose uptake was significantly increased by insulin stimulation, reaching 7.7 times that of the natural water medium without insulin stimulation at 50 ppm, while it was approximately 3.7 times that of the DDW medium without insulin stimulation at the same concentration. (Figure 3a,d). 

Next, to examine the effect of DDW on insulin resistance, myotube-differentiated C2C12 cells were treated with drugs, cultured in medium adjusted with various concentrations of DDW, and then allowed to take up fluorescence-conjugated glucose. To induce insulin resistance, 10 ng/mL TNF-α or 1 μg/mL resistin was added for 24 h. As a result, in a natural water medium, these agents almost completely suppressed the increase in glucose uptake caused by insulin stimulation. In contrast, in DDW medium, these agents had little effect on glucose uptake in the absence of insulin stimulation, and only a slight increase was observed with decreasing DDW concentrations (Figure 3d) before insulin resistance was induced. However, in insulin-stimulated cells, these agents did not sufficiently suppress the uptake of glucose, and at a deuterium concentration of 50 ppm, where the maximum glucose uptake was observed, 45–70% of the glucose uptake before the induction of insulin resistance was confirmed (Figure 3b,c,e,f). These results revealed that DDW attenuated insulin resistance.

In addition, although glucose uptake in the presence of TNF-α was observed throughout the myotubes in the absence of an agent, the effect of resistin was different. Glucose uptake was often detected in only a portion of the myotubes, and myotubes were observed to be divided and atrophied (Figure 3c,f). 

## 4. Discussion

Although there have been reports on the effects of DDW on diabetes, the underlying mechanisms remain unclear. Recent studies using diabetic animal models have reported that increased insulin sensitivity due to the activation of GLUT4 promotes disease improvement, but it is unclear whether DDW has a direct effect on diabetes, since the influence of various other effects of DDW on the body cannot be ignored [13].

In this study, we sought to elucidate the mechanism by which GLUT4 is improved in the disease by directly measuring the effect of DDW on muscle cells in vitro while minimizing the impact of the indirect effects that are believed to occur in the body.

The results revealed that the expression and activation (i.e., membrane translocation) of GLUT4 in C2C12 cells were promoted in the medium adjusted with DDW, and the peaks were near a deuterium concentration of 50 ppm. As the peak of glucose uptake was also near 50 ppm, it is speculated that the increase in GLUT4 gene expression owing to the action of DDW is directly reflected in the increase in the amount of GLUT4 protein, membrane translocation, and glucose uptake. Thus, the most important effect of DDW on GLUT4 may be the promotion of GLUT4 transcription.

Two enhancer domains are known in the promoter region of the human GLUT4 gene, and it is known that GLUT4 enhancer factor (GEF) and myocyte enhancer factor 2 (MEF2) bind to them to control transcription. However, in diabetic mice, the binding ability of MEF2 to the enhancer domain is reduced [27,28].

One hypothesis for the mechanism of the anticancer effect of DDW is that deuterium incorporated into proteins and nucleic acids makes these molecules heavier and stickier, inducing abnormalities in intermolecular interactions, while drinking DDW “lightens” proteins and nucleic acids, normalizing intermolecular interactions [8]. DDW may restore the binding ability of the enhancer domain and transcription factors of the GLUT4 gene by “lightening” them.

This hypothesis leads to the conclusion that the lower the deuterium concentration, the better the gene expression. However, our results indicate that there is a peak concentration of 50 ppm for the expression of GLUT4 and activation by DDW. In in vitro experiments using adipogenic 3T3-L1 cells, we also observed that the expression of GLUT4 and activation reached a maximum at 75–100 ppm (unpublished data), suggesting that the effect of deuterium on the expression of GLUT4 and activation has an optimum concentration that may differ depending on the cell type. The details and causes of the effects of deuterium concentrations below the optimal concentration on cells are currently unknown.

Even if DDW has a strong effect on GLUT4 gene expression, the results of this study showed that the increase in gene expression, protein expression, and membrane translocation was not parallel, suggesting the existence of regulation in the process of translation and membrane translocation (exocytosis), and it is quite possible that DDW also affects these processes. The details of how DDW contributes to the mechanisms of transcription, translation, and membrane translocation remain an important topic for future research on the process of GLUT4 activation. Membrane translocation is particularly important for glucose metabolism, and many cutting-edge studies have demonstrated that one of the causes of insulin resistance is the inhibition of this process [29,30,31,32,33,34].

In this study, two drugs were used to induce insulin resistance in cultured myotubes. Recent studies have reported that adipocytes and related factors secreted by obese enlarged fat cells strongly induce insulin resistance, two of which have been used. TNF-α is an adipocytokine secreted by adipocytes but is also known as an inflammatory cytokine secreted by macrophages [35,36]. Resistin is also an adipocytokine, and excessive secretion of resistin due to obesity is thought to be a cause of diabetes [37]. These agents imparted insulin resistance to myotube-differentiated C2C12 cells and suppressed glucose uptake; however, DDW showed an effect of attenuated insulin resistance. TNF-α is thought to reduce insulin signaling by decreasing the phosphorylation of the insulin receptor substrate-1 (IRS-1) Tyr632 and Akt Ser473, which are downstream effectors of the insulin receptor, thereby resulting in insulin resistance [38,39,40]. The inactivation of the Akt pathway suppresses the membrane translocation of GLUT4 [41]. In contrast, resistin has been reported to suppress GLUT4 [37]. Since DDW attenuates these effects, it is speculated that DDW acts on both the gene expression and membrane translocation of GLUT4.

In addition, these reagents suppress muscle differentiation and induce muscle degradation, such as atrophy, in differentiated C2C12 cells [42,43,44]. At the concentrations used in this experiment, only resistin caused myotube disruption (atrophy), but DDW promoted the uptake of glucose, even in atrophied cells, suggesting that myotube maintenance and GLUT4 function are almost independent and that DDW is only involved in glucose metabolism.

Further detailed in vitro and in vivo studies are required to develop novel DDW-based diabetes treatments.

## 5. Conclusions

A study using cultured muscle cell lines showed that DDW promoted the expression and activation of GLUT4 in muscle cells, with the highest increase occurring at a deuterium concentration of approximately 50 ppm. At the same concentration, the cellular glucose uptake was maximized, and drug-induced insulin resistance was attenuated. These results suggest that DDW is effective in improving diabetes and that an in vitro system is a useful tool for elucidating the detailed underlying mechanisms. 

## Data Availability

The original contributions presented in the study are included in the article, further inquiries can be directed to the corresponding author.
